# The Tomato Variety Affects the Survival of *Shigella flexneri* 2a in Fruit Pericarp

**DOI:** 10.3390/pathogens13050379

**Published:** 2024-05-01

**Authors:** Tania Henriquez, Simona Guerra, Marta Nerini, Diane Purchase, Massimiliano Marvasi

**Affiliations:** 1 Department of Medical Biotechnologies, University of Siena, 53100 Siena, Italy; tania.henriquez@unisi.it; 2 Department of Biology, University of Florence, 50019 Florence, Italy; simona.guerra@edu.unifi.it (S.G.); marta.nerini@unifi.it (M.N.); 3 Department of Natural Sciences, Middlesex University London, London NW4 4BT, UK; d.purchase@mdx.ac.uk

**Keywords:** *Shigella flexneri*, post-harvest tomato, SRL pathogenicity island, vegetable, food safety

## Abstract

The presence of enteric pathogens in produce can serve as a significant means of transmitting infections to consumers. Notably, tomatoes, as a type of produce, have been implicated in outbreaks caused by various human pathogens, such as *Salmonella* enterica and pathogenic *Escherichia* coli. However, the survival characteristics of *Shigella* spp. in tomatoes have not been thoroughly investigated. In this study, we assess the survival of *S. flexneri* 2a in two distinct varieties of post-harvested tomatoes. *S. flexneri* 2a was used to inoculate both regular-sized Vine tomatoes and cherry-type Mini Plum tomatoes. Our findings reveal no significant difference in *Shigella* survival in the pericarp of both varieties on day 2 post-inoculation. However, a significant disparity emerges on day 6, where all recovered *Shigella* colonies exclusively belong to the Mini Plum variety, with none associated with the Vine type. When *Shigella* was inoculated into the locular cavity (deep inoculation), no significant difference between varieties was observed. Additionally, we investigate the potential role of the SRL pathogenicity island (SRL PAI) in the survival and fitness of *S. flexneri* 2a in post-harvested tomatoes. Our results indicate that while the SRL PAI is not linked to the survival of the strains in tomato, it does impact their fitness. These findings underscore the variability in *Shigella* strains’ survival capabilities depending on the tomato variety, highlighting the importance of understanding *Shigella* ecology beyond the human host and identifying molecular determinants influencing bacterial survival to mitigate the risk of future outbreaks. The significance of this data on *Shigella* persistence in fresh vegetables should not be underestimated, as even a small number of *Shigella* cells can pose a threat to the health of individuals.

## 1. Introduction

*Shigella* spp. is a gram-negative rod-shaped bacterium that can cause disease in humans. Four groups are in the genus *Shigella*: *Shigella dysenteriae* (group A), *Shigella flexneri* (group B), *Shigella boydii* (group C) and *Shigella sonnei* (group D) [1] from which *Shigella flexneri* has been identified as the most common cause of bacterial dysentery in developing countries [2,3,4,5,6]. Shigellosis, the term used to denote the illness caused by bacteria belonging to this genus, can be contracted through contamination via the fecal–oral route [1]. It can also be spread by the consumption of water or food contaminated by stools of infected people. In this context, while there is ample understanding that produce can be significantly impacted [7,8], the available information regarding *Shigella* in this regard is limited when compared to *Escherichia coli* or *Salmonella.*

The primary reservoirs of *Shigella* are humans and higher primates [7]. Nevertheless, *Shigella* spp. can survive in different environments; for example, *Shigella flexneri* can survive in distilled water for up to 80 days [9] or in phosphate-buffered saline at pH 7.3 at 5 °C to 10 °C for more than 3 months [10]. Similarly, it has been reported that *Shigella* can survive in urban water [11]. In that study, the authors reported that shigellosis was acquired by drinking tap water, washing utensils in tap water and bathing in tap water [11]. Another study showed that *S. flexneri* could survive for several days at both ambient and refrigerator temperatures when inoculated onto various commercially prepared vegetables [10]. In the case of *Shigella sonnei,* it has also been reported to persist on lettuce for 3 days [12]. Future studies should investigate the molecular mechanisms involved in the survival of *Shigella* outside the human host that remain mostly unknown.

Outbreaks of *Shigella* on vegetables are not common; however, when they occur, they pose serious risks to the infected subjects. A UK outbreak in November 2021 of 17 cases involved MDR *Shigella sonnei*, linked to food from a restaurant chain, with spring onions from Egypt identified as the source [13]. Investigations into the supply chain traced these spring onions back to a sole producer, served across the implicated dining establishments [13].

Another outbreak involved *Shigella sonnei* in April 2018 in England affecting 33 people, linked to food from outlets serving Indian or Middle Eastern cuisine, with coriander as a common factor but no shared supplier identified. Investigations at a specific venue showed that 86% of the affected individuals had consumed dishes that included coriander, either as an ingredient or garnish [14]. Both outbreaks underscore the importance of food chain investigations and the need for improved hygiene practices in cultivation, distribution and food preparation to mitigate public health risks.

Recently, the Health Alert Network (HAN) from the Centers for Disease Control and Prevention (CDC) has posted a health advisory to warn about the increase in extensively drug-resistant (XDR) *Shigella* infections in the US [15]. Similarly, in the EU/EEA, the Annual Epidemiological Report for 2020 about Shigellosis also described the high proportion of resistance to first- or second-line therapy drugs among the isolates tested [16].

In this context, one of the elements that contributes to the multidrug resistance phenotype of *S. flexneri* 2a strains is the SRL pathogenicity island (SRL-PAI) [17,18], as it harbors a 16Kbp region (known as *Shigella* Resistance Locus, SRL) that encodes resistance to streptomycin (*aadA1*), ampicillin (*oxa-1*), chloramphenicol (*cat*) and tetracycline (*tetAR*) [17]. However, little information about other roles of this island, such as metabolic, physiologic or ecological functions, has been published.

Currently, no studies have examined the survival of *Shigella* in various tomato types, despite previous reports of shigellosis outbreaks associated with these fruits [19]. This information is important, since the global tomato market is worth billions of USD annually, with the United States, China, India and Italy being some of the top producers [20,21]. Indeed, according to the Food and Agriculture Organization (FAO) of the United Nations, the world production of tomatoes in 2021 was estimated to be 182.3 million tons [20,21].

While *Shigella* spp. are rarely identified as a source of foodborne illness in developed nations, it is established that the consumption of raw food contaminated with *Shigella* can lead to significant outbreaks [22]. Therefore, we aimed to analyze the proliferation/survival of *S. flexneri* 2a strains in post-harvested tomatoes in this work. We inoculated two tomato varieties, as previous studies on other organisms like *Salmonella* have indicated that replication can vary depending on the tomato genotype. The varieties used were the regular-sized Vine and the cherry-type Mini Plum. We performed experiments emulating the contamination of a superficially damaged tomato (pericarp inoculation) and a heavily damaged tomato (deep inoculation/locular cavity inoculation). Additionally, we also analyzed the contribution of the SRL PAI to fitness and survival. These findings enhance our understanding of *Shigella* survival beyond the human host.

## 2. Materials and Methods

### 2.1. Bacterial Strains and Culture Media

*Shigella flexneri* 2a YSH6000 and *S*. *flexneri* 2a 1363 with a characterized spontaneous deletion of the SRL island were used [17,18]. The strains were cultured in lysogeny broth (LB) medium as described by Bertani (but without glucose) [23] and maintained as frozen glycerol stocks at −80 °C. *Shigella flexneri* 2a YSH6000 was sub-cultured with 10 µg/mL tetracycline (Sigma-Aldrich, St. Louis, MO, USA). Xylose lysine deoxycholate (XLD) (Beckton, Dickinson and Co., Holdrege, NE, USA) and Hektoen agar (Oxoid, Lenexa, KS, USA) were prepared per the manufacturer’s instructions and used for harvesting *S. flexneri* from tomatoes. For the fitness assay, agar plates containing 10 µg/mL tetracycline were used to discriminate between YSH6000 (tetracycline-resistant isolate) and the strain 1363.

### 2.2. Tomato Infection

Bacteria were grown overnight at 37 °C and 200 rpm in LB broth (Fisher Scientific, Lenexa, KS, USA). Then, overnight, the culture was centrifuged at 13,000 rcf for 3 min at room temperature and washed in phosphate buffer saline (PBS, Fisher Scientific) (pH 7.0). This step was repeated three times to remove residual media [24]. Subsequent serial dilutions in PBS achieved a final dilution factor of 10^−5^. This dilution process was designed to yield a target concentration of approximately 100 bacterial cells within a 10 µL volume. The 10 µL sample was then divided into three equal aliquots, each approximately 3.3 µL. Each aliquot was carefully applied to contaminate three superficial wounds, measuring 3 mm each, created on the tomato pericarp, effectively simulating a bacterial infection model. After inoculation, the tomatoes were incubated at 4 °C, 25 °C and 37 °C for 48 (2 days) and 144 h (6 days). When the temperature was set to 4 °C, the tomatoes underwent incubation in a conventional refrigerator equipped with a temperature display. When 37 °C was tested, tomatoes were incubated in a standard incubator. After incubation, tomatoes were macerated in an equal volume of 9.8 g L^−1^ of PBS (Fisher Scientific, Lenexa, KS, USA) using a stomacher (Seward, Bohemia, NY, USA) (200 rpm for 1 min), and the suspensions were plated onto a xylose lysine deoxycholate agar (XLD) or Hektoen agar plate (Oxoid, Lenexa, KS, USA) and incubated at 37 °C overnight. The calculation of proliferation was determined by multiplying the total CFU of each tomato by the dilution factor of the microliter volume plated and by the combined weight of the tomato and the volume of PBS used. This method standardizes the results relative to the size of individual tomato. The increase in proliferation was calculated by dividing the total CFU recovered from each tomato by the total CFU inoculated into each fruit. The following formula was used: Log_10_ × (total CFU_harvest_/total CFU_inoculum_). To take into account the limit of detection of 0 and the not possible application of the Log_10_ scale, all CFU values were summed by 0.1. The increase in proliferation was further subjected to the log_10_ transformation.

Mini Plum (cherry-size, average weight 18.5 ± 2.6 (g) and Vine (average weight 58.5 ± 11.5 g) tomatoes were purchased at the local grocery store. Tomatoes were washed three times with distilled water and dried. Each experiment was performed with six biological replicas. No signs of spoilage were observed after 6 days of incubation.

**Figure 1:**
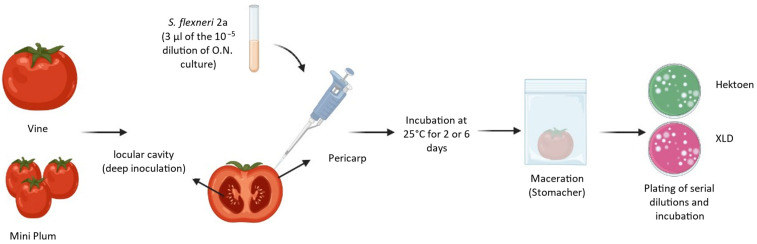
A schematic illustration of the procedure for the analysis of the persistence of *Shigella* strains in post-harvested tomato fruits. Mini Plum and Vine tomato varieties were used to inoculate *S. flexneri* 2a strains using pericarp or locular cavity inoculation. After 2 or 6 days of incubation at 25 °C (in this figure) but also 4 °C and 37° were tested. The tomatoes were macerated in a bag using a stomacher, and a serial dilution of the resulting suspension was plated in a media selective for *Shigella* isolation (Hektoen or XLD). Created with BioRender.com.

For experiments using deep inoculation (locular cavity), bacterial inoculum was prepared following the same procedure used for pericarp infection. Using a micropipette, 3 μL of the 10^−5^ dilution was put inside the locular cavity of each tomato (three inoculations per tomato; Figure 1). The incubation was performed at 25 °C for 144 h (6 days). Finally, the tomatoes were macerated in a stomacher in an equal volume of 9.8 g L^−1^ of PBS, and the suspensions were plated onto an XLD or Hektoen agar plate and incubated at 37 °C overnight. Proliferation/persistence was calculated as explained before.

The inoculation process is summarized in Figure 1.

### 2.3. Fitness of Shigella flexneri Strains

To calculate a competitive index, wild-type *S. flexneri* 2a YSH6000 (tetracycline-resistant) and *S. flexneri* 2a 1363 with the deleted SRL island (tetracycline-sensitive) were inoculated at a 1:1 ratio into tomatoes (approximately 10^5^ CFU/mL were inoculated). The relative ratios of the strains in the inocula and the recovered samples were calculated by plating the bacterial suspension in media with and without tetracycline (in parallel). The competitive index was calculated for each treatment using the formula Log_10_[(R_out_/S_out_)/(R_in_/S_in_)], where R is the number of resistant colonies, and S is the number of sensitive cells in the initial inoculum (in) or in the recovered samples (out). The log-transformed values of the competitive index are presented. The statistical and biological significance of each competitive index was established by comparing the log values of the competitive indices of each pair to the log of the competitive index similarly calculated for the Vine variety and Mini Plum variety. Up to twelve biological replicas were performed for each fitness experiment.

### 2.4. Statistical Analysis

The statistical software SSPS package 29.0.2.0 and GraphPad/Prism 9 were used to perform the *t*-test, considering statistical significance at a *p*-value of <0.05.

## 3. Results

While there is knowledge that certain enteric pathogens, like *Salmonella*, can colonize and thrive in tomato fruits [25,26,27], the understanding of *Shigella*’s role in this context is significantly limited. In this context, we aimed to analyze the growth/survival of *S. flexneri* 2a in two varieties of tomato. To that end, the tomato Vine variety (bigger size) and Mini Plum (cherry-type size) were used. Post-harvested Vine and Mini Plum tomatoes were infected with about ~100 cells of *S. flexneri* 2a. Cells were harvested at 2 (48 h) and 6 days (144 h) of incubation at three different temperatures 4 °C, 25 °C and 37 °C to simulate the infection of a post-harvest tomato stored in the fridge, room temperature and a hot temperature (such as summertime).

**Figure 2:**
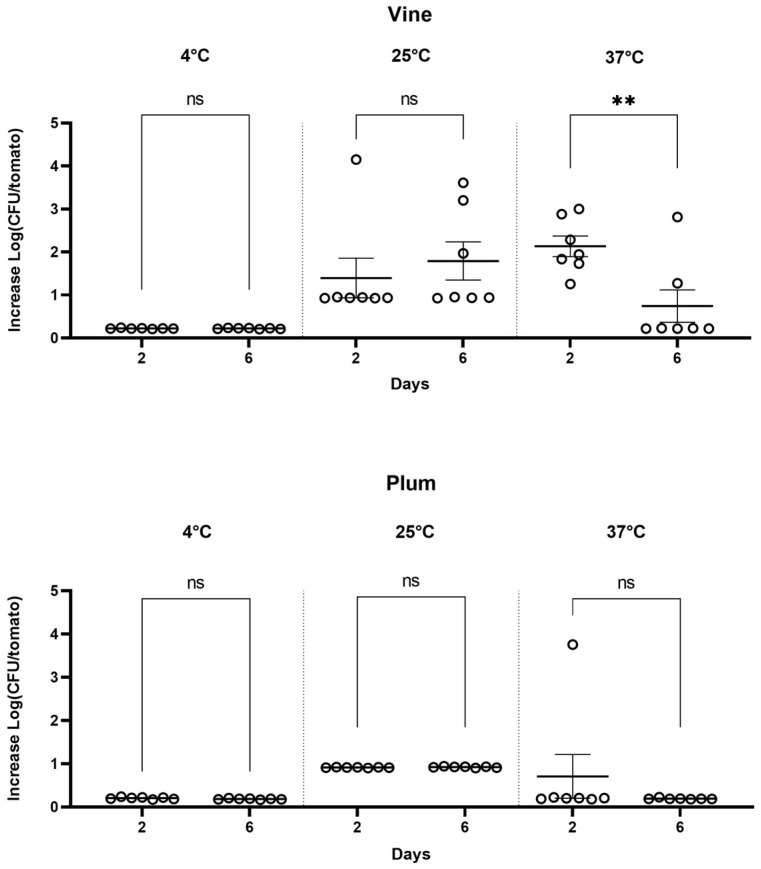
*Shigella* proliferation in tomato varieties Vine and Plum at different days and different temperatures. Circles represent biological replicas. Horizontal line is average of increase in proliferation. Error bars are standard error. (** *p* < 0.0021). ns: not significant.

*Shigella* exhibited generally poor growth in tomato pericarp. *Shigella* colonies were recovered from some tomatoes at both time points, but their proliferation was extremely limited. Notably, the Vine variety demonstrated a marked increase in *Shigella* proliferation at 25 °C, with an approximate 2-log rise on average (Vine, Log CFU 1.4 ± 0.4) after 2 days and was maintained over the 6 days of the experiments (Vine, Log CFU 1.7 ± 0.4). Always in Vine at 37 °C by day 6, *Shigella* colonies were no longer isolated (Vine, from Log CFU 2.1 ± 0.2 day 2 to 0.7 ± 0.3 day 6), suggesting that the reduction in CFUs might be attributed to the wilting of the tomatoes. In the Plum variety, at the temperatures of 4 °C and 37, the analysis of the growth across different days revealed that the inoculation of *Shigella* in the tomato pericarp showed no major differences after 2 or 6 days of incubation (see Figure 2. Also, for this variety, 25 °C was the temperature at which *Shigella* replicated the most.

**Figure 3:**
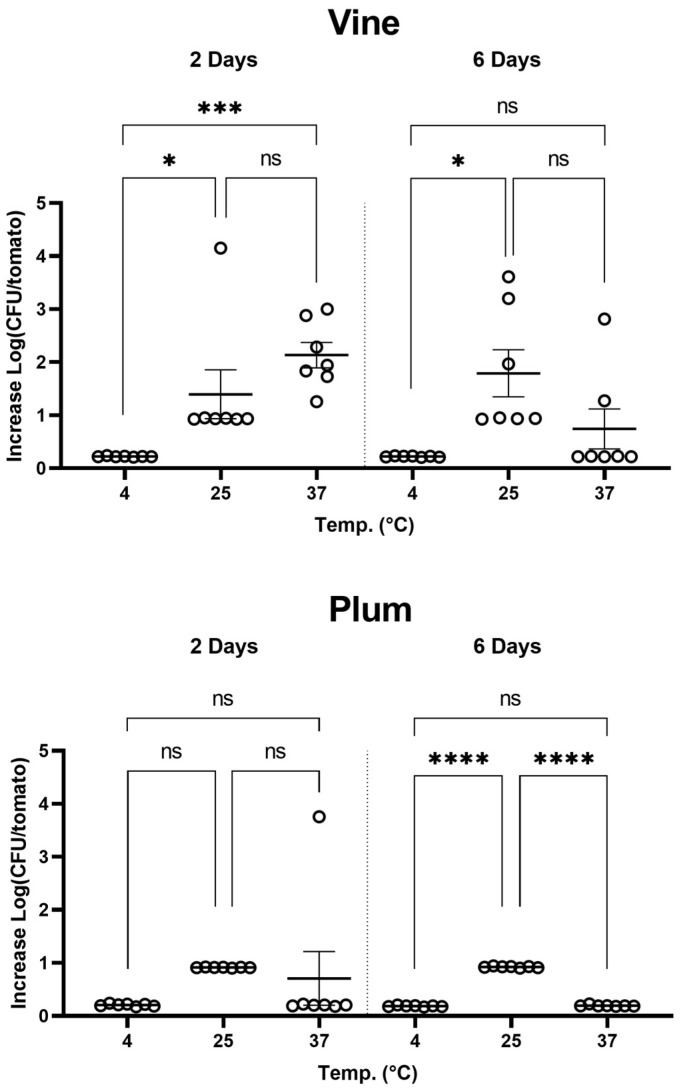
Temperature-dependent *Shigella* proliferation in tomato varieties Vine and Plum. Circles represent biological replicas. Horizontal line is average of increase in proliferation. Error bars are standard error. (* *p* = 0.0332); (*** *p* = 0.0002); (**** *p* < 0.0001). ns: not significant.

The impact of temperature on *Shigella* survival is shown in Figure 3. As anticipated, no growth occurs at 4 °C, confirming the bacterium’s mesophilic metabolism that prevents replication at low temperatures. Contrastingly, at 25 °C, both the Vine and Plum varieties exhibited significant CFU recovery, indicating this to be the most conducive temperature for *Shigella* survival and a critical point for storage risk assessment. Incubation at 37 °C generally prevented CFU recovery. The most at risk was Vine stored for 2 days at 25 °C and 37 °C where the highest CFU recovery was noted (Log CFU 2.1 ± 0.2). In conclusion, the data suggest that 25 °C and 2 days of storage is the optimal combination of temperature and time for *Shigella* survival, making it a crucial factor for consideration in the storage of the Vine variety. As previously seen for *Salmonella*, the variety of tomato is also a central factor that fosters the proliferation of enteric pathogens.

**Figure 4:**
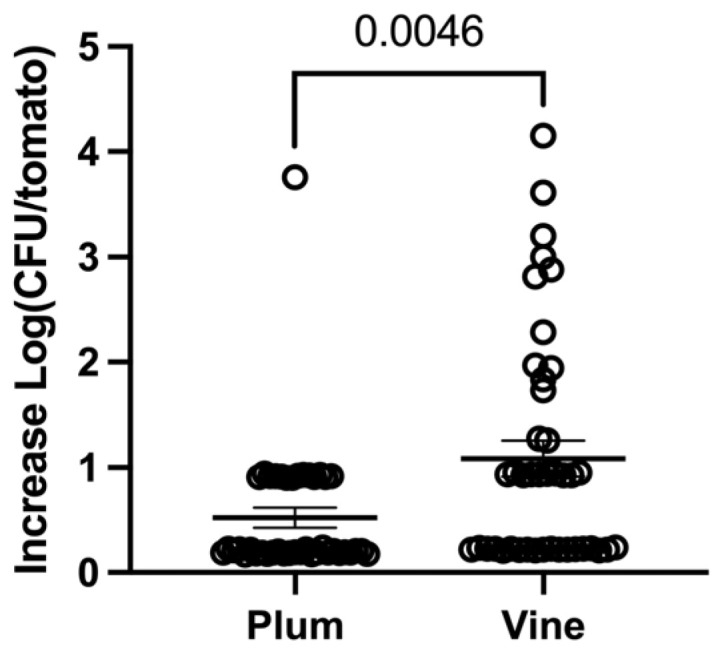
Overall *Shigella* proliferation in tomato varieties Vine and Plum. Circles represent biological replicas. Horizontal line is average of increase in proliferation. Error bars are standard error.

To determine the effect ascribed to the varieties, all experiments were summarized to determine an overall effect of the variety in all conditions. The variety Vine (Log CFU 1.1 ± 0.1) had the highest recovery among all samples tested, when compared with Plum (0.5 ± 0.1), Figure 4.

Also, it is important to note that the survival of *S. flexneri* was limited to a few tomatoes (biological replicas). Most of the selective Petri plates showed 0 recovery of *Shigella* colonies. The recovery in the Plum genotype was low; about 1% were recovered in pericarp. On the other hand, in 13% of the Vine tomatoes, *Shigella* was counted in pericarp. Despite the relatively low recovery of *Shigella* in fruit, when the bacteria were able to adapt and find a suitable microenvironment, a potentially dangerous survival was measured.

Additionally, the survival of *Shigella* strains in tomatoes was tested using deep inoculation in the gel contained in the locular cavity. The recovery was negligible in both tomato varieties, due to the highly acidic environment [28].

**Figure 5:**
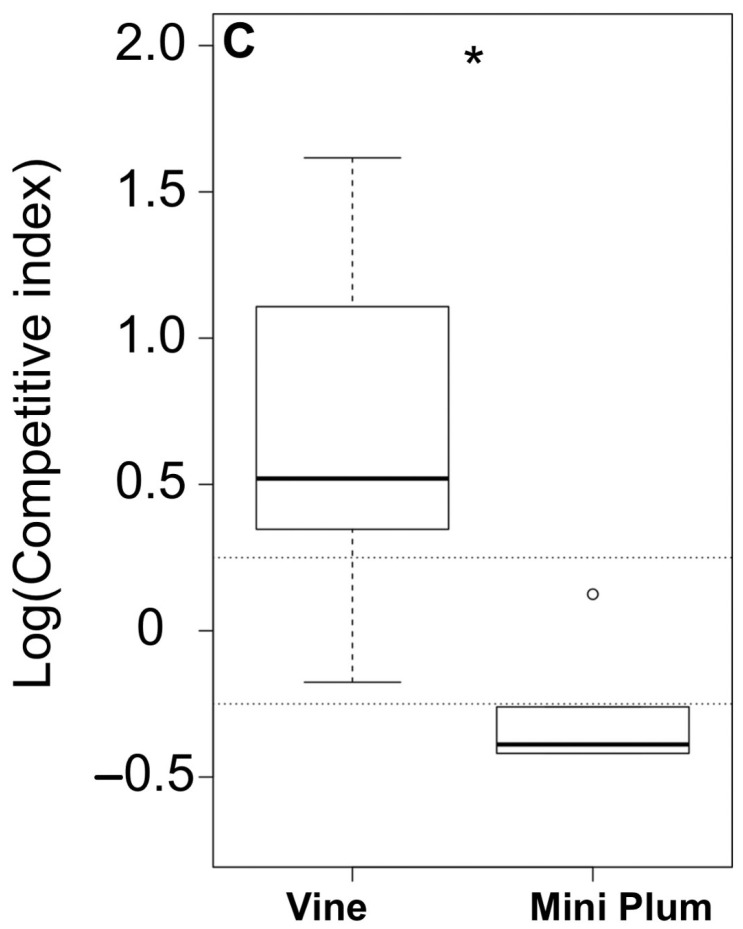
The role of the SRL PAI in the fitness of *S. flexneri* 2a strains in tomato fruits. The fitness test *S. flexneri* 2a YSH6000 (marked with the tetracycline-resistance cassette) was co-infected in a 1:1 ratio with *S. flexneri* 2a 1363 into the pericarp. The ratios of the wild type/mutant in the inoculum and in the recovered tomatoes were estimated by patching on selective media after 48 h of incubation. Values above 0 indicate that *S. flexneri* YSH6000 is outcompeting the mutant strain (or surviving longer). On the other hand, values below 0 indicate that *S. flexneri* 2a 1363 is outcompeting the wild type (or surviving longer). When the value is close to 0, no advantage in terms of fitness is measured. Dashed lines estimate a cut-off beyond which changes in competitive fitness are biologically significant. The box-plots encompass the lower and upper quartiles, thick lines within the box are the median values and the whiskers indicate the degree of dispersion of the data. (* *p* < 0.05). Outliers are shown as dots.

Genomic islands have been previously associated with the ability of bacteria to survive in specific environments [29]. The SRL PAI is one of the islands of *S. flexneri* 2a, and it has been recently associated with the ability to metabolize D-aspartic acid (which could be useful to survive outside the human host) [30]. In this context, we decided to analyze the association of the SRL PAI with the survival of *S. flexneri* 2a in the pericarp of tomato. *S. flexneri* 2a YSH6000 (SRL PAI-positive strain) and *S. flexneri* 2a 1363 (YSH6000-derivative strain with a deletion of the SRL PAI) survival in the pericarp of tomato was compared. Our results indicate that there is no association between the presence of the island and the survival in both varieties of tomato; however, although not significant, we did observe a trend. Therefore, we decided to perform a fitness test with both strains in tomato. Our results indicated that during the co-infection of the *Shigella* pair in Vine tomato, *S. flexneri* 2a YSH6000 was outcompeting the strain *S. flexneri* 2a 1363 (Figure 5). Conversely, in Mini Plum cherry tomatoes, the absence of the SRL PAI conferred a fitness advantage to the *S. flexneri* 2a 1363 strain (Figure 4). These results suggest that in *Shigella* spp., specific islands or genetic components could differently affect the colonization of produce.

## 4. Discussion

Shigellosis is a worldwide disease that could be associated with the intake of contaminated food and water. Studies have shown that *Shigella* can contaminate various food sources, including vegetables, meat, egg products and dairy products, highlighting the importance of understanding the mechanisms behind its persistence and virulence in food [6,7,31,32]. Shigella’s ability to survive in refrigerated foods and its ease of transmission make it a significant concern in foodborne outbreaks, especially in settings where food requires handling during preparation [22]. Furthermore, the presence of *Shigella* in food samples, including post-harvest tomato, has been demonstrated, indicating that food can serve as an environmental source for the spread of different virulence genes among *Shigella* isolates [33].

In 2005, an outbreak of shigellosis caused by *S. flexneri* 2a showed that sliced tomatoes were able to retain viable *S. flexneri* up to 72 h after exposure to the bacterium [19]. However, the survival of this organism in food remains largely uncharacterized in comparison to other enterobacteria.

Thus, in this work, we studied the survival of *S. flexneri* 2a in post-harvested tomatoes (Mini Plum and Vine varieties) and the contribution of the SRL pathogenicity island on bacterial persistence and fitness in tomato pericarp.

Our results showed that *S. flexneri* exhibits minimal growth in tomato pericarp, which is a clear contrast with other enterobacteria, especially *Salmonella* [34]. The interactions between *Shigella* and plants can be assumed to be transient or opportunistic. In fact, we found that *Shigella* cannot proliferate in contrast to *Salmonella* [35] in 6 days (*Salmonella* can reach up to a Log CFU 10^5^ increase in proliferation in just 3 days).

In this context, we also report the presence of *Shigella* strains in the pericarp of tomato after 144 h post-inoculation in Vine at 25 °C, a much longer survival/persistence when compared with the 72 h measured by Reller and colleagues (2006) but in other varieties. It is also important to note that bacterial survival in sliced tomato (e.g., the sample collected by Reller and colleagues) is expected to be shorter when compared with pericarp inoculation, due to the exposure of *Shigella* to the acidity of the tomato juice. In contrast, pericarp proliferation is expected to be intercellular, and bacteria would not be exposed to organic acids [36].

Our results showed that the survival/persistence of the *Shigella* strains was different depending on the variety of the tomato and the site of inoculation. Other studies documented changes in the ability of crops and crop genotypes to support different populations of enteric pathogens [25,37,38,39]. The fluctuation of persistence according to the tomato genotype has been previously studied in other enteric bacteria. The expression of specific *Salmonella* genes has been proved to respond to the tomato genotype and plant metabolites [25,35].

Temperature also showed an effect in *Shigella* persistence on tomatoes. We found that 25 °C is the temperature that exposes the consumer to higher risk (Figure 3). With reference to the experiments at 4 °C, *Shigella* can persist in food even at low temperatures like 4 °C but without replication. The persistence of *Shigella* in food at 4 °C is concerning due to its low infectious dose, making it easily transmissible through contaminated food and water [40]. *Shigella* is known to survive in refrigerated foods, especially those that are served cold or raw and require handling during preparation [22].

In addition to the persistence according to the tomato genotype, we showed that the SRL pathogenicity island also contributed to the fitness of *S. flexneri* 2a strains.

We previously mentioned that the SRL PAI encodes for resistances to streptomycin, ampicillin, chloramphenicol and tetracycline. Our experiments showed that the presence of such antibiotic cassettes is not necessarily an energetic burden for the cell and that high-nutrient food, like a ripe tomato, can support the persistence of this antibiotic multidrug-resistant strain. Contrastingly, in different tomato genotypes, such as the Mini Plum variety, the removal of the island would yield a positive impact on fitness. This is attributed to the elimination of the resource-intensive machinery associated with antibiotic resistance genes and other systems that appear unnecessary in these conditions, such as the Ferric Dicitrate Transport System (Fec). These results are in agreement with the current literature that shows that the tomato genotype plays a pivotal role in the persistence of *Enterobacteriaceae* in post-harvested tomato [34].

The characterization of the ecology of *Shigella* in its natural niches is important to understand how *Shigella* can survive outside the human body. Since it was described that as few as 10 cells can cause shigellosis [41], the finding that *S. flexneri* 2a could survive and be recovered from tomatoes after 6 days post-inoculation is concerning. In this context, further studies need to be performed to clearly determine the maximum amount of time that *Shigella* can survive in a specific type of food and to establish which role other environmental factors play such as temperature and humidity [42].

*Shigella* can reach tomatoes through a number of ways: the interaction of infected food handlers with produce, which seems to be the greatest risk, irrigation via unsafe water (e.g., polluted freshwater) and fertilization with non-composted manure. *Shigella* has been found in manure and irrigation water, posing a potential risk to human health. Studies have indicated the presence of *Shigella* in manure samples [43] and in surface waters, including irrigation water, due to fresh contamination by fecal material, especially in regions with poor sanitation practices [44]. The presence of *Shigella* in manure and irrigation water is concerning as it can lead to the transmission of the pathogen to crops and subsequently to humans through the consumption of contaminated produce or water [43]. Furthermore, the detection of *Shigella* in manure samples highlights the potential for the pathogen to persist in the environment and serve as a reservoir for further contamination [45]. The proper management of manure and irrigation water is crucial to prevent the spread of *Shigella* and reduce the risk of foodborne illnesses. Implementing measures to treat manure and ensure the quality of irrigation water can help minimize the presence of this enteric in agricultural settings [45]. Additionally, promoting good agricultural practices and monitoring water sources for microbial contamination are essential steps in safeguarding public health from the risks associated with *Shigella* contamination in manure and irrigation water. Moreover, some strain of *Shigella* isolated from manured agricultural clay soils and slurry samples showed sulfonamide resistance genes in a study over a 2-year period [45]. The SRL PAI is involved in the spread of multiple-antibiotic resistance in *Shigella* species, highlighting its importance in the context of antimicrobial resistance [46]. Additionally, the SRL PAI contributes to the multidrug resistance phenotype observed in *Shigella flexneri* strains (Henríquez et al., 2020). Studies have demonstrated the existence of *Shigella* in food samples, indicating that these isolates with different virulence genes could serve as environmental sources for epidemic spread [22]. The SRL PAI has been identified in numerous *Shigella* strains, emphasizing its significance in the epidemiology and resistance profiles of these bacteria [46].

It would also be important to study to what extent agronomic practices contribute to limit the pre- and post-harvest contamination and proliferation of human pathogenic *Shigella* spp. in vegetables [27]. Additionally, further environmental studies should focus on to what extent *Shigella* spp. contaminate plants according to environmental conditions on the field (e.g., previous work has been conducted with *Salmonella*) [26].

In conclusion, our results indicated that *S. flexneri* 2a exhibits minimal proliferation in tomatoes; however, it survived up to 6 days in the superficially damaged Vine variety. Also, our results showed that the SRL PAI does not have a role in the survival of *S. flexneri* 2a, but it affects the fitness of the strains depending on the tomato variety.

Altogether, these results highlight the importance of characterizing the ecology of *Shigella* outside the human host and the relevance of identifying the molecular determinants of bacterial survival to efficiently avoid future outbreaks.

## Data Availability

The data will be made available by the authors on request.
